# Supplementary material for: Oropharyngeal mucosal transmission of Zika virus in rhesus macaques
Source: Nat Commun. 2017 Aug 1;8:169. doi: 10.1038/s41467-017-00246-8 (PMC5539107; doi:10.1038/s41467-017-00246-8)
Supplement: Supplementary file 1 — Supplementary Information Supplementary [file 41467_2017_246_MOESM1_ESM.pdf]

File name: Supplementary Information
Description: Supplementary Figures and Supplementary Table

File name: Peer Review File
Description:

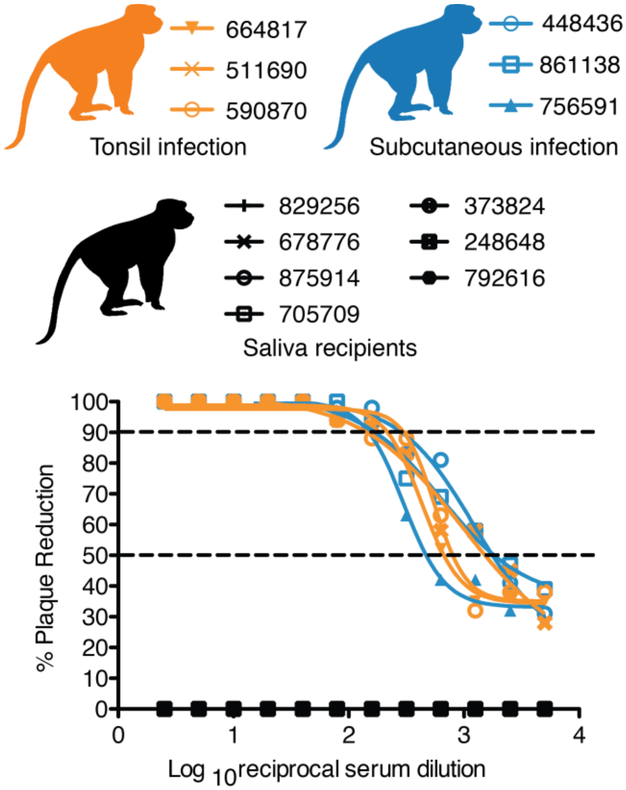

Supplementary Figure 1. Neutralization by ZIKV immune sera from tonsil inoculated and subcutaneously inoculated macaques. Immune sera from macaques inoculated subcutaneously (blue), inoculated with the virus stock via the tonsils (orange), or inoculated with donor saliva via the tonsils, conjunctiva or nasal passage (black) were tested for their capacity to neutralize ZIKV-FP. ZIKV was mixed with serial 2-fold dilutions of serum for 1 hour at 37˚C prior to being added to Vero cells. Infection was measured by plaque reduction neutralization test (PRNT) and is expressed relative to the infectivity of ZIKV-FP in the absence of serum. The concentration of sera indicated on the x-axis is expressed as Log10 (dilution factor of serum). The dilution of sera at half-maximal neutralization of infection (EC50) was estimated by non-linear regression analysis and were 2.803 (861138), 3.081 (448436), 2.479 (756591), 2.638 (664817), 2.991 (511690), and 2.746 (590870). Neutralization curves for each animal at 28 dpi are shown.

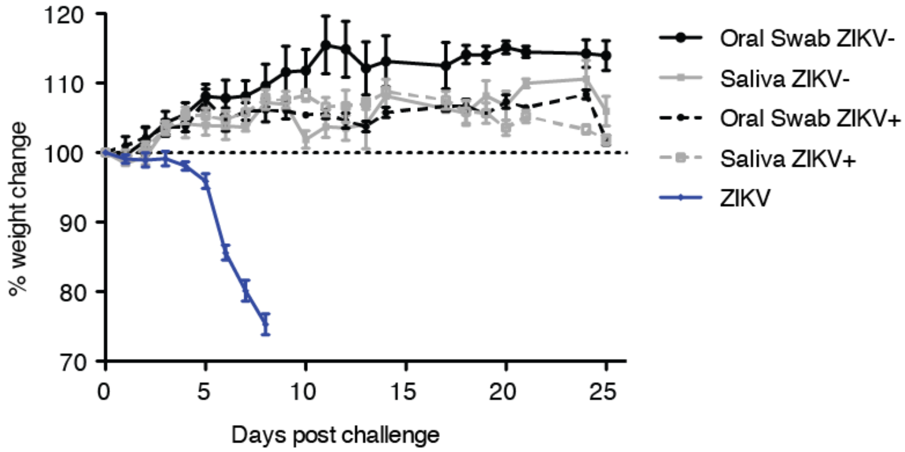

Supplementary Figure 2. Injection of ZIKV+ oral swab eluate or ZIKV+ saliva does not cause mortality or morbidity in IFNAR-/- mice. IFNAR-/- mice (n=3 per group) were inoculated in the left hind footpad with ZIKV+ or ZIKV- oral swab eluate (black) or ZIKV+ or ZIKV- saliva (gray). Mice were monitored until 25 dpi; all survived without signs of morbidity. Changes in weight were calculated daily for ZIKV+ and ZIKV- oral swab eluate and ZIKV+ or ZIKV- saliva inoculated mice. Lastly, 7 IFNAR-/- mice were inoculated with the stock ZIKV-FP virus (blue) and all 7 mice were humanely euthanized at 8 dpi because they met the criteria for euthanasia. Error bars represent the standard deviation of the mean.

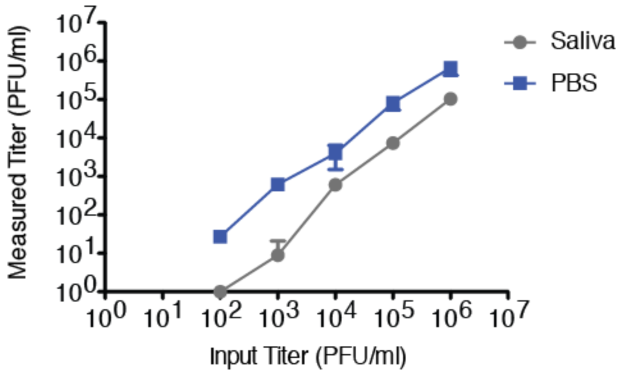

Supplementary Figure 3. Saliva reduces the infectivity of ZIKV in Vero cells. ZIKV was mixed with saliva collected from uninfected macaques in a 1:10 or 1:3 dilution for 1 hour at 37C and added to Vero cells at final concentrations of 1 x 10^2 - 1 x 10^5 PFU and 8 x10^5 PFU respectively. Infection was measured by plaque assay. Phosphate buffered saline spiked with the same concentrations of ZIKV were used as positive controls. Error bars represent standard deviation of the mean from two independent titration experiments.

Supplementary Table 1. Results for samples tested for infectivity by plaque assay on Vero cells

Sample Type	*Animal ID*	*Viral Load (copies/mL)*	*Days Post Infection*	*Plaque Assay Result*
Oral swab	448436	2,770	7	-
Saliva	861138	11,200	6	-
*Oral swab**	678776	<100	97	-
*Saliva**	678776	<100	97	-
ZIKV-FP stock	N/A	3.9×10^9	N/A	+++

*Samples collected from a saliva recipient (678776) that served as negative controls.

- No observable plaques.

+++ Plaques too numerous to count.
